# Sequential efgartigimod and ofatumumab for stiff-person syndrome: a case report illustrating a pathophysiology-informed strategy and literature review

**DOI:** 10.3389/fimmu.2026.1811565

**Published:** 2026-06-26

**Authors:** Chenxi Yang, Junqiu Zhou, Haishan Jiang

**Affiliations:** 1 Department of Neurology, The People’s Hospital of Baiyun District, Guangzhou, China; 2 Department of Neurology, Nanfang Hospital, Southern Medical University, Guangzhou, China

**Keywords:** anti-GAD65, B-cell depletion, efgartigimod, FcRn blockade, ofatumumab, sequential immunotherapy, Stiff-Person Syndrome

## Abstract

**Background:**

Stiff-person syndrome (SPS) is a rare, antibody-mediated autoimmune disorder of the central nervous system, most frequently associated with antibodies against glutamic acid decarboxylase 65 (GAD65). Management of patients with an insufficient response to conventional immunotherapy remains a clinical challenge.

**Case presentation:**

We report a 40-year-old woman with anti-GAD65 antibody-positive SPS. She experienced severe relapse and functional disability (modified Rankin Scale [mRS] score 4) despite plasma exchange and maintenance immunosuppression. We designed a sequential, mechanism-base regimen: the neonatal Fc receptor antagonist efgartigimod (3 cycles) was administered to rapidly reduce pathogenic IgG, followed by the anti-CD20 monoclonal antibody ofatumumab (2 cycles) to deplete B-cells and provide sustained immunomodulation.

**Results:**

This sequential strategy resulted in substantial and sustained clinical improvement (mRS score 4 to 1, Barthel Index 75 to 100) and a reduced clonazepam requirement despite persistently high anti-GAD65 antibody titers, demonstrating efficacy beyond mere antibody reduction. The regimen was well-tolerated with no significant adverse events.

**Conclusion:**

This case provides preliminary clinical experience with a sequential efgartigimod-ofatumumab strategy in treatment-resistant SPS. This approach, which combines rapid antibody clearance with sustained B-cell suppression, not only affirms the role of ofatumumab in long-term immunomodulation but also highlights the potential synergy of sequential administration, warranting further investigation.

## Introduction

Stiff-person syndrome spectrum disorder (SPS) represents a severe central nervous system autoimmune disease mediated by antibodies targeting inhibitory synaptic proteins (1). First-line immunotherapies, such as intravenous immune globulin (IVIg) (2) and plasma exchange (PE) (3), aim to clear pathogenic antibodies but yield transient or inadequate responses in some patients. For cases with an inadequate response, therapies targeting the cellular source of autoantibodies have been explored as a rational therapeutic approach (4).

We present the case of a patient with anti-glutamic acid decarboxylase (GAD) 65 antibody-positive SPS with significant disability who did not achieve sustained disease control despite PE and concurrent immunosuppressive therapy. Efgartigimod is neonatal Fc receptor (FcRn) antagonist that has shown efficacy in myasthenia gravis (5, 6) and other antibody-mediated diseases (7, 8), and it is currently the only FcRn inhibitor approved in China. Ofatumumab is a fully human anti-CD20 monoclonal antibody (9), chosen to minimize the risk of anti-drug antibodies. To achieve the dual goals of rapid symptom control and sustained immune modulation, we implemented a sequential strategy using two agents with complementary mechanisms: First, efgartigimod was administered to rapidly reduce IgG levels (5, 6), followed by ofatumumab to induce profound B-cell depletion (9–11). The strategy aimed to achieve rapid clinical improvement through the former and sustain long-term efficacy via the latter. This report details the clinical course and outcomes of this pathophysiology-informed approach, alongside a review of the relevant literature.

## Case presentation

### Medical history and diagnosis

A 40-year-old female patient was transferred to our institution in April 2025 presenting with “progressive lower limbs stiffness accompanied by paroxysmal painful spasms over 5 years, with worsening symptoms in the past 2 months”.

A diagnosis of SPS was clinically established in November 2021. This conclusion was reached based on the combination of: 1) classic progressive axial and limb rigidity with stimulus-sensitive painful spasms; 2) highly elevated anti-GAD65 antibody titers in both serum and cerebrospinal fluid (CSF) (1:10,000 and 1:1,000, respectively; detected by Cell-based transfection immunofluorescence assay [CBA]) while glycine receptor and amphiphysin antibodies were negative; and 3) exclusion of alternative diagnoses. Although the initial electromyography did not demonstrate continuous motor unit activity—a finding atypical for classic SPS—the overall clinical and serological profile was deemed sufficiently characteristic to warrant the diagnosis and initiate immunotherapy (12, 13).

Her clinical status was tracked using a combination of disease-specific semi-quantitative scales (2, 14, 15) and functional measures. After a course of PE, her symptoms significantly improved: the distribution-of-stiffness index (from 2 to 1; lower score indicates less stiffness) and the heightened-sensitivity scale (from 2 to 0; lower score indicates fewer spasms). Her overall disability, measured by the modified Rankin Scale (mRS), improved from 3 to 1 (lower score indicates better function). She was subsequently maintained on clonazepam (0.5 mg every 8 hours) and mycophenolate mofetil (1 g twice daily), remaining clinically stable for approximately three years.

### Severe relapse and pre-treatment evaluation

In March 2025, the patient’s symptoms deteriorated rapidly. Lower limb stiffness progressed to the point of wheelchair dependence, with spasticity easily triggered by sound or mild pain stimuli, resulting in severely limited mobility (stiffness score 4, heightened-sensitivity score 5, and mRS 5). Serum and CSF anti-GAD65 antibody titers retested at an external hospital and were both 1:100 (anti-GlyR and anti-amphiphysin remained negative). A second course of PE was administered but failed to provide satisfactory sustained improvement. During treatment, the patient developed new-onset neck pain, confirmed by vascular ultrasound as a jugular vein thrombosis. Following consultation with vascular surgery and the referring institution, therapeutic anticoagulation with rivaroxaban (15 mg once daily) was initiated for 6 months.

Following transfer to our institution in April 2025, neurological examination revealed an alert patient with fluent speech. Cranial nerve testing was unremarkable, with full extraocular movements, smooth saccades, and absence of nystagmus, diplopia, or oscillopsia. Assessment of muscle strength in the extremities was precluded by spasticity. Notable findings included increased lower limb spasticity, paraspinal hypertonicity, and spontaneous painful spasms. Deep tendon reflexes were brisk in the lower limbs, with bilateral Babinski and Pussep signs present. Functional scores were as follows: stiffness score of 3, heightened-sensitivity score of 5, modified Rankin Scale (mRS) score of 4, and Barthel Index of 75 (range 0-100, with higher scores indicating greater independence in activities of daily living).

### Sequential therapy and clinical course

Based on the patient’s insufficient and unsustained improvement with prior therapies, we initiated a targeted treatment regimen. On April 19, 2025, she received the first intravenous infusion of efgartigimod 800 mg (10 mg/kg). The following day, she experienced several episodes of generalized rigidity with severe pain, which were fully controlled by April 22 following intensive therapy with intravenous diazepam and sedatives (dexmedetomidine, midazolam). Her symptoms subsequently improved, and maintenance therapy with oral clonazepam (2 mg every 8 hours) was continued.

On May 9, 2025, the patient received a second 800 mg infusion of efgartigimod. A pre-infusion CSF anti-GAD65 antibody titer was 1:100. During this hospitalization, the patient also received a course of IVIg (2 g/kg total). Given the primary goal of achieving long-term disease suppression and the limited durability of antibody-clearing therapies alone, we initiated subcutaneous ofatumumab (20 mg) as the maintenance therapy on May 18, followed by a gradual tapering of the clonazepam. At the follow-up in July 2025, the CSF anti-GAD65 titer remained at 1:100.

Following the initial treatment sequence, the patient maintained a sustained remission for several months. In August 2025, a manageable clinical fluctuation occurred, with all clinical scores deteriorating (as detailed in **Table 1**), prompting a timely therapeutic reinforcement. A third 800 mg infusion of efgartigimod was administered on August 29, followed by a second dose of ofatumumab on September 6, which promptly re-established disease control. Upon discharge on September 6, her maintenance regimen was consolidated with clonazepam 2 mg every 12 hours, which she continued thereafter. Although she was instructed to return within 2–3 days for peripheral blood lymphocyte subset analysis to assess B-cell depletion, the patient did not comply. Following the completion of pharmacotherapy, she commenced community-based rehabilitation.

**Table 1:** Dynamic changes of key clinical metrics during treatment.

Timepoint (key event)	Stiffness score	Heightened-sensitivity score	mRS score	Barthel index	Clonazepam dose	CD19+B cells (%)	Major therapeutic event
2021-11-07	2	2	3	Not assessed	4mg q8h	Not tested	Diagnosis, pre-treatment
2021-11-21	1	0	1	Not assessed	0.5mg q8h	Not tested	After 1st PE
2025-03-24	4	5	5	Not assessed	2mg q8h	Not tested	Relapse, pre 2nd PE
2025-04-18	3	5	4	75	2mg q8h	Not tested	Pre-squential therapy after admission
2025-04-26	2	0	2	100	2mg q8h	Not tested	After 1st Efgartigimod
2025-05-23	1	0	1	100	Tapering started	7.24 to 1.05	After 2nd Efgartigimod+1st Ofatumumab
2025-08-27	2	2	3	95	1mg q8h	7.39	Symptom fluctuation, pre-3rd Efgartigimod
2025-09-06	2	0	1	100	2mg q12h	4.47	Pre-2nd Ofatumumab (prior to administration and discharge)

The CD19+ B-cell percentages shown were obtained from clinical flow cytometry analysis (Guangzhou Huayin Medical Laboratory Center).

### Follow-up and functional outcome

**Figure 1:**
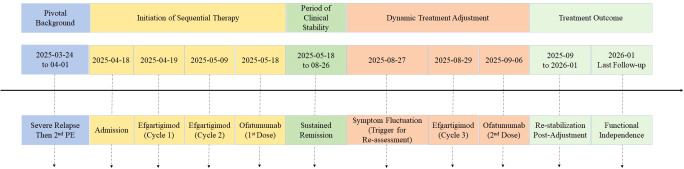
Timeline of key therapeutic decisions and clinical events. The diagram highlights the sequential administration of efgartigimod and ofatumumab, and the dynamic treatment adjustment (augmented efgartigimod followed by ofatumumab) made in response to a clinical fluctuation. PE, plasma exchange.

At the last follow-up in January 2026, the patient reported no recurrence of painful spasms, with largely normal gait. She had successful return to work, and could perform complex activities such as hiking and yoga. Her maintenance regimen consisted of oral clonazepam (2 mg every 12 hours) and mycophenolate mofetil (1 g twice daily). Objective functional measures confirmed a remarkable recovery: an mRS score of 1 and a Barthel Index of 100 were maintained, indicating complete functional independence. Notably, the CSF anti-GAD65 antibody titer remained persistently positive at 1:100 throughout the observation course, despite marked clinical fluctuations (see **Table 2** for detailed chronological profile). **Figure 1** illustrates the patient’s clinical course timeline, highlighting the sequential treatment strategy and its dynamic adjustment in response to disease activity.

**Table 2:** Chronological anti-GAD65 antibody testing profile.

Date	Sample	Anti-GAD65 antibody titer	Testing laboratory & location	Assay method	Reference range
2025-03-24 (After Relapse)	Serum	1:100	Cred Diagnostics (Hangzhou)	CBA	Negative
2025-04-03 (Post-2nd PE)	Serum	1:100	Cred Diagnostics (Hangzhou)	CBA	Negative
2025-05-09 (Pre-1st efgartigimod)	CSF	1:100	Kindstar (Guangzhou)	CBA	Negative
2025-07-06 (Period of Clinical Stability)	CSF	1:100	Kindstar (Guangzhou)	CBA	Negative

CBA, Cell-based transfection immunofluorescence assay.

### Safety monitoring

The treatment was well-tolerated with no clinical infections or other significant adverse events reported. Laboratory monitoring showed the expected rapid pharmacodynamic effect of ofatumumab, with the peripheral blood CD19+ B-cell percentage (the standard clinical marker for total B-cell counts) decreasing from 7.24% pre-injection to 1.05% on the third day post-injection in May 2025. Despite persistently seropositive anti-GAD65 titers, the serum total IgG level measured in September 2025 (after completion of three cycles of efgartigimod and prior to the second ofatumumab infusion) was 7.87 g/L, slightly below the institutional lower limit of normal (8.6 g/L). All routine laboratory parameters, including complete blood counts, serum chemistry, and coagulation profiles remained stable and within normal limits throughout treatment period. Pre-existing anemia did not progress. Although an elevated D-dimer level was observed, it showed a declining trend over time while remaining above the normal range. Key clinical metrics are summarized in **Table 1**.

## Discussion

### 1. Current management and therapeutic challenges in stiff-person syndrome

SPS is a rare autoimmune neurological condition characterized by impaired GABAergic neurotransmission, leading to progressive axial and limb rigidity, painful spasms, and neurological hyperexcitability (1). At initial presentation (2021), the patient lacked the classic electromyography finding of continuous motor unit activity, which was a key criterion in earlier diagnostic frameworks (12, 15). The diagnosis process in this case highlights the clinical heterogeneity of SPS, reaffirms that its diagnosis heavily relies on characteristic clinical manifestations and serological detection of specific autoantibodies such as anti-GAD65 (1, 2, 12, 16). Current management of SPS is based on immunosuppressants and symptomatic antispastic drugs, with IVIg and PE as rescue therapies during acute exacerbations (2, 3, 17). Although the anti-CD20 monoclonal antibody rituximab has been widely used in SPS, its efficacy remains inconsistent across studies (18–20), and some patients show no response or experience relapse after initial treatment (12, 21). This therapeutic challenge is exemplified by public accounts from well-known individuals, such as the renowned singer Céline Dion’s disclosed long-term struggle with severe, refractory SPS, underscoring the significant disease burden posed by functional disability and limited treatment options for a subset of patients. Reflecting this unmet need, recent clinical research has diversified, exploring novel agents targeting specific immune pathways (see **Table 3** for an overview of recent investigational approaches). Hence, the management of SPS largely relies on expert experience and case reports data, highlighting the urgent need to develop pathophysiology-based, precision treatment strategies that extend beyond conventional frameworks.

**Table 3:** Overview of recent investigational approaches in stiff-person syndrome (based on ClinicalTrials.gov).

NCT number	Intervention	Phase	Status	Mechanism of action
NCT06588491	Biological: Standard lymphodepletion regimen	2	Active, not recruiting	Intervention of autoimmune response
NCT06703333	Combination Product: Extracorporeal photopheresis (ECP)	Early 1	Not yet recruiting	Immunoregulation
NCT06242678	Device: spinal cord stimulation (SCS) trial lead	Not Applicable	Completed	Targeting components of neuronal inhibitory signaling pathways in spinal cord
NCT02282514	Biological (Autologous Hematopoietic Stem Cells) (47)	4	Terminated	Maximal suppression of the immune system
NCT06375993	Drug: ADI-001, Fludarabine and Cyclophosphamide	1	Recruiting	Intervention at the root of the autoimmune response
NCT07341828	Biological: CD20/BCMA-directed CAR-T cells	1	Not yet recruiting	B-cell depletion
NCT06528392	Biological: Efgartigimod	2	Not yet Recruiting	FcGn antagonist (IgG clearance)
NCT03829826	Biological: subcutaneous immunoglobulin therapy (SCIg) (48)	Not Applicable	Unknown	Pathogenic antibodies clearance

All trials can be accessed on the U.S. National Institutes of Health ClinicalTrials.gov registry using the provided NCT identifier.

### 2. FcRn inhibition in SPS: sustained clinical benefit amidst stable antibody titers

A pivotal observation is the patient’s marked clinical improvement following FcRn inhibition despite persistently stable CSF anti-GAD65 antibody titers. Previous trials in myasthenia gravis and across several other antibody-mediated diseases have generally correlated clinical improvement with reductions in both total serum IgG and pathogenic antibody titers (6–8, 22). Documented evidence also supports the clinical benefit of efgartigimod in patients with SPS (23). The observed dissociation between symptom relief and static antibody titers aligns with reports from other antibody-mediated disorders, such as anti-NMDAR encephalitis, where clinical improvement might precede the clearance of CSF antibodies (24). More specifically, longitudinal studies in SPS have proposed that GAD65 IgG-secreting B-cell clones may persist within the intrathecal compartment, requiring only minimal clonal recruitment or expansion—potentially from long-lived plasma cells or T-cell-dependent plasmablasts—to maintain antibody levels (25). This persistent intrathecal humoral activity likely underlies why anti-GAD antibody titers are unreliable biomarkers of disease activity in SPS (17, 18, 21). The pharmacology of efgartigimod, which accelerates IgG catabolism via FcRn blockade rather than suppressing synthesis (5, 22), provides the direct mechanistic basis for symptomatic relief without an immediate decline in titers, and underscores the primacy of clinical assessment. The subnormal serum total IgG level, measured below the normal range, further corroborates this mechanism.

Although direct quantitative comparison between serum and CSF titers is not valid in our case, the qualitative persistence of a positive titer in both compartments underscores the stability of the humoral immune response throughout the clinical course. The patient’s prompt response to efgartigimod re-administration suggests that symptom recurrence was driven by humoral activity from either a persistent reservoir (26, 27) (e.g., long-lived plasma cells, which are not directly targeted by anti-CD20 therapy) or repopulating B-cell clones (or even, theoretically, newly emergent ones) (28). The stable antibody titers throughout are consistent with the postulated ongoing intrathecal antibody production by a maintained cellular reservoir. It is recognized that the pathogenic potential of an autoantibody is finely regulated by factors beyond mere titer, such as its epitope specificity and binding affinity (23, 29, 30). While our single case cannot delineate these precise mechanisms, it robustly illustrates that clinical benefit from efgartigimod in SPS can be decoupled from static antibody titer measurements. This finding argues against over-reliance on serial antibody titers as a sole biomarker of treatment response in SPS and highlights the need to identify more dynamic biomarkers of disease activity and therapeutic effect.

### 3. Rationale for sequential therapy and clinical course

Ofatumumab induces deep, sustained B-cell depletion with a favorable safety profile, offering a viable therapeutic option for neuroimmune diseases (10, 31–41). The process of B-cell depletion triggers immunodynamic changes, including B-cell activating factor (BAFF) elevation that supports B-cell survival and differentiation (26, 42). Notably, transient autoantibodies rises post-rituximab have been linked to clinical flares in other diseases (26, 43).

We hypothesize a sequential synergy: FcRn inhibition rapidly reduces IgG and immune complex levels, thereby alleviating Fc gamma receptors (FcγR) saturation on effector cells (44). This may mitigate any potential pro-inflammatory effects of this early post-depletion phase, a period of complex immunodynamic adjustment where rapid B-cell clearance can transiently alter the cytokine milieu (e.g., elevating BAFF) (26) and may contribute to immune activation. As anti−CD20 antibodies clear B cells primarily via FcγR-dependent mechanisms such as antibody-dependent cellular cytotoxicity (ADCC) (9, 44), this prior “conditioning” by FcRn blockade may promote a more stable clinical course.

In our case, clinical fluctuation coincided with CD19+ B-cell repopulation (7.39%) after initial induction. This observation aligns with the established pattern in B-cell depletion therapy, where clinical relapse has been temporally linked to B-cell repopulation in other conditions (45, 46). The observed B-cell rebound may reflect an early post-depletion adjustment phase. Our dual intervention simultaneously targeted both antibodies and their cellular source. Thus, the combination may achieve both immediate control and durable remission by mechanistically bridging complementary immune pathways.

### 4. Strengths, limitations and future perspectives

This single-case report provides preliminary experience with a sequential strategy for treatment-resistant SPS. It describes a combination not previously reported in SPS, provides mechanistic insights through clinical and immunological data, and contextualizes the case within current SPS therapies via a literature review. Its findings, while illustrative, require validation in controlled trials and cannot be generalized. Several limitations should be acknowledged. As the patient received mycophenolate mofetil alongside IVIg administered simultaneously during hospitalization, and though no immediate benefit was noted at that time, the possibility of subsequent efficacy cannot be ruled out. However, the subsequent improvement in symptoms was temporally correlated with the sequential treatment. This underscores a common limitation in evaluating novel strategies in refractory cases, where multimodal therapy is often employed pragmatically. Furthermore, the assessment of the immunological impact was limited by incomplete serial monitoring and methodological constraints. First, B-cell measurements were reported as percentages rather than absolute counts, which limits direct comparison with studies employing absolute quantitation. Second, post-ofatumumab B-cell counts were not consistently obtained, and only a single post-efgartigimod serum IgG level was measured, precluding analysis of its dynamic reduction and recovery. These gaps limit a precise analysis of the pharmacodynamic interaction and temporal profile of combined agents.

Future prospective research should incorporate a standardized serial immune-monitoring protocol of both humoral (e.g., IgG levels) and cellular (e.g., B-cell subsets) parameters at defined timepoints relative to each treatment cycle. This is crucial to precisely characterize the dynamic immunologic effects of this sequential approach, and identify biomarkers correlating with clinical efficacy.

## Conclusion

This report details the successful management of an SPS patient with inadequate response to prior immunotherapies using a sequential efgartigimod-ofatumumab strategy based on complementary mechanisms. The strategy demonstrates clinical potential, and the dynamic changes observed during treatment provide valuable insights that may inform future precision therapy approaches in SPS. This case offers valuable clinical and theoretical reference for future exploration of mechanism-guided treatment pathways in antibody-mediated neurological diseases.

## Data Availability

The raw data supporting the conclusions of this article will be made available by the authors, without undue reservation.
